# Optimization and Identification of Antioxidant Peptide from Underutilized* Dunaliella salina Protein*: Extraction,* In Vitro* Gastrointestinal Digestion, and Fractionation

**DOI:** 10.1155/2019/6424651

**Published:** 2019-08-20

**Authors:** Enqin Xia, Lu Zhai, Zhigang Huang, Hairong Liang, Hui Yang, Gang Song, Weiqiao Li, Huanwen Tang

**Affiliations:** Dongguan Key Laboratory of Environmental Medicine, School of Public Health, Guangdong Medical University, Dongguan 523808, China

## Abstract

DPPH• scavenging peptides (<3kDa) from underutilized* Dunaliella salina* protein were obtained by the following successive treatment, i.e., ultrasound extraction, simulated* in vitro *gastrointestinal digestion hydrolyzation, and membrane ultrafiltration classification. The optimal condition for ultrasound-assisted extraction was an ultrasound wave with 800 W of power treating a mixture of 60 mL of 1.0 mol L^−1^ NaOH and 2 g algae powder for 15 min. A high correlation (r=0.8146) between DPPH• scavenging activity and yield of the intact peptides showed their antioxidant capacity. Simulated* in vitro *digestion assay resulted in excellent DPPH• scavenging activity of the total peptide, amounting to (86.5 ± 10.1)%, comparing with the nondigestion samples at (46.8 ± 6.5)%. After fractionation, the 500-1000 Da fraction exhibited the highest DPPH• scavenging activity (81.2 ± 4.0)%, increasing 1.5 times due to digestion. Then, the 500-1000 Da fraction was analyzed by RPLC-Q Exactive HF mass spectrometer, and 4 novel peptides, i.e., Ile-Leu-Thr-Lys-Ala-Ala-Ile-Glu-Gly-Lys, Ile-Ile-Tyr-Phe-Gln-Gly-Lys, Asn-Asp-Pro-Ser-Thr-Val-Lys, and Thr-Val-Arg-Pro-Pro-Gln-Arg, were identified. From these amino acid sequences, hydrophobic residues accounted for 56%, which indicated their high antioxidant property. The results indicated that underutilized protein of* Dunaliella salina* could be a potential source of antioxidative peptides through simulated* in vitro* gastrointestinal digestion.

## 1. Introduction

Nowadays, it was recognized that the growing chronic diseases linked to the key biological molecules such as DNA, protein, and lipid were disposed by the excess reactive oxygen and free radicals species in the body due to variation of life style and environment problem [1–4]. An effective measure to decrease and prevent this damage has been regarded as inhibiting and removing the free radicals through the reaction of natural antioxidant compounds [5, 6]. Recently, according to many epidemiological and empirical studies, bioactive peptides derived from various protein sources have showed great potential to scavenge the free radicals, and the highest activity was observed at fractions with molecular weight less than 3 kDa [7–10]. Therefore, the characteristics of peptides with antioxidant activity less than 3 kDa have been paid increasing attention of researchers in relative field.

The peptides from marine microalgae, such as* Chlorella, Arthrospira,* have also gaining attention as new antioxidative alternatives in the last few years [11–16].* Dunaliella salina*, as a unique unicellular microalgae, is rich in good proteins and can survive in various salt concentrations (0.2%-35%) and light intensity conditions. It is inferred that dramatic changes in living conditions can bring massively unique bioactive proteins and peptides different from those of terrestrial plants and other microalgae [17–19]. Fortunately, some researchers have confirmed that, due to salt stress and flagellar disassembly, peroxiredoxin mRNA and protein expression significantly increased in* Dunaliella salina* cells [20–22], while salt stress has also been found to contribute to accumulation of unique bioactive proteins [23]. Thus, the information seemingly gives some light on the antioxidant activities of protein and peptide derived from this microalgae. However, main attention about* Dunaliella salina* was paid to the bioactive compounds, such as astaxanthin, *β*-carotene, polyphenol, and so on. There is limited information available in the literature on the bioactivity of isolated protein from* Dunaliella salina* or it hydrolyzed peptides and even less for its scavenging free radical or antioxidant activity. Therefore, further research is needed to better understand and assess the peptides from this special microalgae for their abilities to scavenge free radicals to prevent chronic diseases.

Recently, centrifugal ultrafiltration, combined with dialysis, has been regarded as an efficient approach to purify and fractionate the bioactive proteins or peptide in very small volume, which will be verified in this work [24–26]. Ultrasound assisted extraction has been considered as an efficient and green/ecofriendly technology to disrupt the matrices and subsequently release the target component by bubble cavitation at mild condition in short time [27, 28]. Meanwhile, it has been reported that the technique of enzymolysis could be employed to search the new and small molecular peptide with stronger DPPH• scavenging ability and other bioactivities [29, 30]. In addition, whether the stability of bioactivity was retained during the gastrointestinal digestive juice might be a large block for preparation of peptide with marked bioactivities practical application in human.

In present study, obtained by a combination approach of ultrasonic wave, stimulated* in vitro* gastrointestinal digestion, and ultrafiltration centrifugation, the DPPH• scavenging activity of the peptide released from* Dunaliella salina* was investigated. The peptide less than 3 kDa with strong DPPH• scavenging activity would also be identified by RPLC-Q Exactive HF mass spectrometer. This study is to highlight the significant antioxidant activities of the peptide exploration from underutilised marine microalgae protein sources.

## 2. Materials and Methods

### 2.1. Chemicals

1,1-Diphenyl-2-picrylhydrazyl (DPPH•), pepsin (3640 U mg^−1^ protein), trypsin (3640 U mg^−1^ protein), acarbose, and ascorbic acid (VC) were purchased from Sigma (St. Louis, MO, USA). All other chemicals used in the experiments were of analytical reagent grade from Sangong Biotech Co., Ltd. (Shanghai, China).

### 2.2. Methods

#### 2.2.1. Sample Preparation

*Dunaliella salina *(freeze-dried powder) was supplied by Wenzhu-Biotech Co. Ltd. (Xi'an, China) during January and February, 2017. The algae powder (2 g) was crushed in mortar and blended with 60 ml 1.0 mol L^−1^ sodium hydroxide solution with a magnetic stirrer. The homogenate was treated with ultrasonic device (Kunshan Ultrasonic Instrument Co. LTD., China) to release the intracellular proteins at 25°C for 30 min. The mixtures were centrifuged at 12,000 rpm for 5 min at 4°C. Peptides with 100-10000 Da molecular weight were collected and freeze-dried for detection of the content and DPPH• scavenging activity of peptide. According to the results, the optimal extraction conditions of protein, such as the concentration and volume of sodium hydroxide solution, the time, power, and temperature of ultrasound radiation, were obtained.

#### 2.2.2. Simulated In Vitro Gastrointestinal Digestion

Simulated* in vitro* gastrointestinal digestion has been previously conducted for the optimal extract of algae protein according to He et al. [31] with some modification. Briefly, the total protein of microalgae freeze-dried powder was extracted at optimal extraction conditions and precipitated at pI by settling overnight using HCl at the concentration of 5 mol L^−1^. After centrifugation and freezing out, 1.0 g of protein dissolved in a 100 mL of PBS was mixed with 0.1 mL of solution containing 0.20 (g mL^−1^) commercial pepsin (3000 U g^−1^ protein) in normal saline (0.15 mol L^−1^ NaCl), which was incubated for 4 h at 37°C. Then, the pH was adjusted to 6 by 0.1 mol L^−1^ NaOH. Then, 1.00 mL of a trypsin solution (2 g mL^−1^, 0.1 mol L^−1^ NaHCO_3_, pH = 6) preheated at 37°C was added and incubated at 37°C for 4 h. Finally, trypsin was inactivated by heating at 80°C for 15 min. After that, the digestion solution was centrifuged at 5°C, 11,000 rpm for 10 min. The supernatant was conducted.

#### 2.2.3. Fraction of the Peptide

The peptide in crude extract and digestion solution were desalted and fractionated using a combined dialysis and ultrafiltration-centrifugation technique. Briefly, the digestion peptide solution was dialyzed using a dialysis tube with a molecular weight cut-off (MWCO) of 100 Da in distilled water with three changes for 2 h each. A total of 10 ml of desalted peptide was pooled into the 50 mL ultrafiltration centrifuge tubes with molecular weight cut-off (MWCO) of 500, 1000, 3000 Da (Millipore, USA), in turn. After centrifugation for 30 min (4500×g), 4 fractions of permeated peptide were collected. These samples were then saved for further content and bioactivities studies.

#### 2.2.4. Quantitation and Identification of the Peptide

The peptide concentration in samples (crude extract,* in vitro* digestion, and fractions) was detected by the bicinchoninic acid (BCA) assay. The reaction reagents were added according to the BCA Protein Assay Kit (Yuangye Biotech CO., Shanghai, China). The absorbance was measured at 562 nm using a microplate reader (PerkinElmer, Shanghai, China) after incubation for 30 min. Bovine serum albumin (0.01 - 0.15 mgmL^−1^) was used as a standard.

The fraction of digested 500 to 1000 Da with strong DPPH• scavenging activity was subjected to an Acclaim PepMap reversed-phase liquid chromatography (RPLC) with an C18, 3*μ*m, 100Å (75*μ*m i.d. ×150mm) column coupled online with a Q Exactive HF mass spectrometer (Thermo Fisher Scientific, Waltham, MA, USA). Herein, analysis and identification were done according to Narváez-Rivas et al. [3] with a slight modification. For separation part, solvent composition of the mobile phase at the two channels was 0.1% (v/v) formic acid and 2% (v/v) acetonitrile for channel A and 0.1% (v/v) formic acid and 80% (v/v) acetonitrile for channel B. After desalting and cleanup, 5 *μ*L of sample was loaded on a pre-column (Acclaim PepMap RPLC C18, 5 *μ*m, 100Å, 300 *μ*m i.d. × 5mm) before analysis column and eluted at a flow rate of 300 nL/min. The peptides were separated by a gradient elution from 6% to 9% B in 8 min, to 14% B in 16 min, to 30% B in 36 min, to 40% B in 15 min, and to 95% B in 3 min. Then, according to Shan et al. [32] with a slight modification, all MS experiments were performed in positive and negative ion modes using a heated electrospray ionization source. The source and ion transfer parameters applied were as follows: spray voltage 3.5 kV (positive) and 2.8 kV (negative). For both ionization modes, the sheath gas, aux gas, the capillary temperature, and the heater temperature were maintained at 35, 15 (arbitrary units), 325°C, and 300°C, respectively. The S-Lens RF level was set at 50. The Orbitrap mass analyzer was operated at a resolving power of 70,000 in full-scan mode (scan range: 350–1800 m/z; automatic gain control (AGC) target:3e6) and of 17500 in the Top 10 data dependent MS2 mode (stepped normalized collision energy: 25 and 30; injection time: 250 ms; isolation window: 1.0 m/z; AGC target: 1e5) with dynamic exclusion setting of 10.0 s.

#### 2.2.5. DPPH• Free Radical Scavenging Effect

The scavenging effect of each fractionated peptide was carried out by DPPH• method of He et al. [31] with some modifications. Aliquots of each peptide were dissolved in methanol (20−660 *μ*g mL^−1^, final concentration). A 100 *μ*L of peptide sample (pH 6.86 modulated by phosphate buffer) was added into 100 *μ*L of DPPH radical solution in ethanol (0.125 mmol L^−1^).The mixture was blended thoroughly and incubated at room temperature for 30 min in the dark. Then, the absorbance was monitored on microplate reader (PerkinElmer, Shanghai, China) at 517 nm until a plateau was reached. Ascorbic acid (VC), as a positive control, was determined in a concentration range of 0–0.5 mmol L^−1^. The absorbance of the phosphate buffer solution (pH 6.86) substituting for sample and ethanol for DPPH solution was defined as control for total and sample, respectively. Percent DPPH• scavenged was calculated by

(1) $$\begin{array}{c} \%I=\frac{\left(A_{control1}-\left(A_{sample}-A_{control2}\right)\right)}{A_{control1}}*100 \end{array}$$

where A_control1_ is absorbance of DPPH• methanol solution and A_sample_ is absorbance of test compound. Tests were carried out in triplicate and average.

#### 2.2.6. Statistical Analysis

The colorimetric measurement tests were performed in triplicate. The measurement results are shown as the mean ± SD in the dose-response curves. A* P* value of less than 0.05 was considered to be statistically significant. Statistical analyses were performed using the software SPSS for Windows version 17.0 (SPSS Inc., Chicago, IL, USA). All of the linear regression analyses described in this paper were processed using the Origin Professional software (2017 version, OriginLab, Northampton, MA, USA).

## 3. Results and Discussion

### 3.1. Extraction and Optimization of Peptide from the Crude Extract

**Figure 1:**
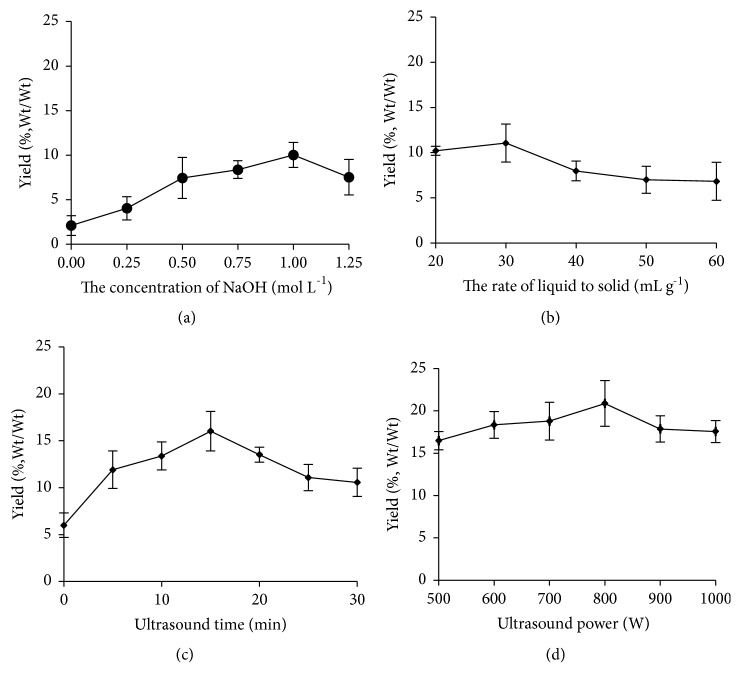
Extraction and optimization of peptide from the crude extract.

In literature, the peptide of* Chlorella* extracted with hot water had been found possessing notable inhibition of MMP-1 expression in skin fibroblasts [12]. It indicated that some small molecular proteins from microalgae can also exhibit appreciable biological activities without protease hydrolysis. In present paper, optimization of the ultrasound assisted extraction conditions of the peptide from* Dunaliella salina* was firstly investigated according to the yield of crude protein at first. And the NaOH solution was used as extraction solvent due to the pI of proteins from* Dunaliella salina from* different saline at 4.7-6.0 [33]. The extraction parameters, i.e., the concentration and volume of extraction solvent and the power and time of ultrasound, were optimized by single factors test. The results were illustrated in Figure 1.

Figures 1(a) and 1(b) displayed effect of the concentration and volume of NaOH solution on extraction efficiency. Aided by an ultrasound wave at 500 W of power radiating for 20 min, the yields of peptide significantly increased modestly (0-1 mol L^−1^) with increasing in the concentration of NaOH, and the same variation tendency was observed for liquid to material (20-30) mL g^−1^. The NaOH concentration of 1 mol L^−1^ and the liquid to material of 30 mL g^−1^ gave the highest value (10.0 ± 1.1)% and (11.1 ± 1.2)%, respectively. The alkali solution was also suitable for extracting peptide from seaweed* Ascophyllum nodosum*, compared to acid assisted extractions at comparable concentrations [34]. This may be explained due to alkaline conditions facilitating the solubilization of water insoluble hydrophobic peptide [35]. Then, it was observed that as the concentration of NaOH is higher than 1.0 mol L^−1^ (Figure 1(a)) or liquid to material is up to 30 mL g^−1^ (Figure 1(b)), the peptide yield notably declined. Maybe, protein was hydrolyzed into amino acids or aggregated by more NaOH. Therefore, 60 mL of 1.0 mol L^−1^ NaOH was used as extraction solvent for 2 g algae sample for the next test.

The impact of ultrasound power and time on the peptide yield was illustrated in Figures 1(c) and 1(d). A significant increase of peptide yield was observed with prolonged ultrasonic time from 0 to 15 min. The highest peptide yield was observed at 15 min of ultrasonic radiation (16.0 ± 1.2)%. Then the yield decreased with further radiating up to 30 min (Figure 1(d)). The similar observation was also noted for soy peptide solution [36]. In addition, a slight increase of peptide yield was found with the power rising to 800 W, and then it declined as the power increased to 1000 W. The highest peptide yield was (20.9 ± 2.7)% at 800 W. According to literature, low intensity ultrasound is commonly used to extract and evaluate the physicochemical properties of foods, and appropriately prolonging treatment time or increasing the radiation intensity to sufficient energy can break the covalent bonds or hydrogen bonds and release target molecules from material [37–39]. However, the decrease of yield of peptide by continuously prolonging treatment time or enhancing the power might be due to the denaturation and aggregation of peptide caused by excessive ultrasonic radiation [40, 41]. Therefore, an ultrasound wave radiating at 800W of power for 15 min was employed to extract peptide for next experiments.

### 3.2. Effect of Ultrasound Radiation on the DPPH• Scavenging Activities of Peptide

**Figure 2:**
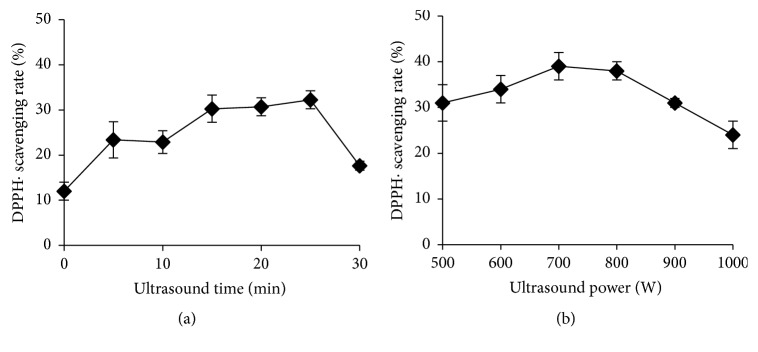
Effect of ultrasound radiation on the DPPH• scavenging activities of peptide.

In literature, the model of scavenging the stable DPPH radical is widely used to evaluate the antioxidant activities of natural compounds [42]. Herein, under the optimal solvent conditions obtained in the previous step, effect of time and intensity of ultrasound on the DPPH• scavenging activities of extraction peptide from* Dunaliella salina* was investigated. The results were displayed in Figures 2(a) and 2(b).

From Figure 2(a), at 500 W of ultrasound power, the DPPH• scavenging rate of extracted peptide increased with prolonging the ultrasound radiation time from 0 to 25 min. The highest level of DPPH• scavenging activity amounted to (32.3 ± 2.2)%. Then, as treatment continued to 30 min, the DPPH• scavenging rate obviously decreased. In addition, different increasing trends were found from 0 to 25 min. At first, from 0 to 5 min, a notable increase of the rate was in a large margin, which means that the ultrasonic treatment was an efficient and optimum scheme for extraction of peptide with antioxidant activity. However, a slight increase of the DPPH• scavenging activity was found with ultrasonic time varying from 10 to 15 min. The values from 5-10 min and 15-25 min nearly hold the line. The results suggest that ultrasound wave could accelerate the establishment of an equilibrium for dissolution of the peptide with DPPH• scavenging activity among algae cell and the extraction solvent in a short time, even at 5 min, which is huge advantage as an extract projection for bioactive compounds compared to the conventional solvent extraction. However, target peptide could be degraded after a long exposure to ultrasonic radiation, causing the antioxidant activity to decrease. The same results were found in author previous reports [43].

From Figure 2(b), with the ultrasonic power increasing from 500 to 800 W, the DPPH• scavenging rate slightly increased. However, using severe ultrasound wave with ultrasonic power over 800 W, the DPPH• scavenging rate of peptide notably decreased. The same variety trend has been explained as severely ultrasonic radiation could destroy the primary structure of target compounds by effectively breaking out the intramolecular chemical bonds, and too small pieces of proteins may lose this free radical scavenging activity [41, 43]. Therefore, the ultrasonic radiation at 800 W for 15 min at the optimal conditions for the peptide yield could also obtain the highest level of DPPH• scavenging activity.

**Figure 3:**
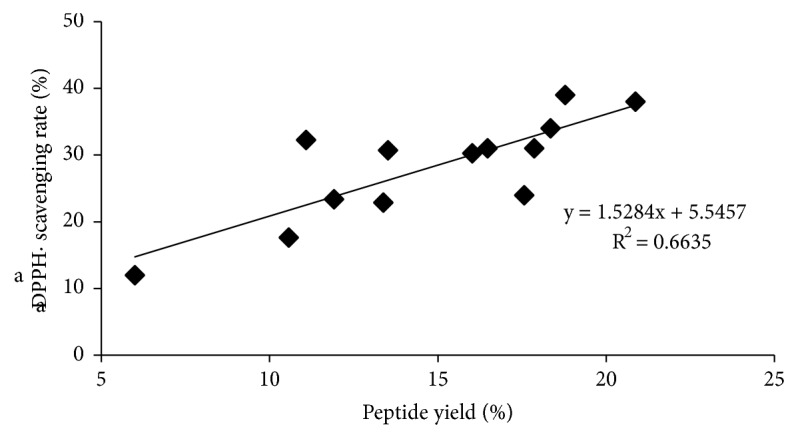
The relationship of the yield and DPPH• scavenging activity of peptide.

In addition, the correlation of DPPH• scavenging rate and total peptide content was analyzed to confirm protein acting as the main active ingredient in extract samples. The results was displayed in Figure 3. From Figure 3, a considerably positive correlation (r = 0.8146) between the DPPH• scavenging rate and total protein content indicated that protein could be the component responsible for free radical scavenging ability of these crude protein extracts [44]. However, all the samples exhibited poor DPPH• scavenging rate less than 40% in present study.

### 3.3. Effect of In Vitro Simulated Digestion on the Contents and DPPH• Scavenging Activity of Peptide

Herein,* in vitro* simulated gastrointestinal digestion was used for enzymolysis of the protein obtained from* Dunaliella salina* by above optimal scheme to search peptide with high DPPH• scavenging activity. The results were illustrated in Table 1.

From Table 1, at the same contents of extraction peptide, the DPPH• scavenging rate increased near twice, over 86.5%, by subjecting to* in vitro* simulated gastrointestinal digestion comparing to the intact peptide. Similarly, a notable increase was seen in the capacity to scavenge free radicals and reduce ferric ion of whey protein isolates due to simulated* in vitro* gastrointestinal digestion compared with the intact samples [29]. A significant increase in the antioxidant activity was also observed for the fermented donkey milk isolate after simulated* in vitro* gastrointestinal digestion [30]. These results indicated that simulated* in vitro* gastrointestinal digestion might be employed as an efficient scheme to optimize and search for the target peptide with high antioxidant activity. In addition, from Table 1, the large antioxidant activity with DPPH• scavenging rate indicated the peptide from* Dunaliella salina* might be a potentially functional and pharmaceutical ingredient to deal with oxidant stress in human through common oral administration. Therefore, the particular peptide being respondent to the DPPH• scavenging activity was interesting in the next study, and its molecular weight was investigated by fractionation technique.

### 3.4. Effect of Fractionation on the Yield and DPPH• Scavenging Capacities of Peptide

According to Olagunju et al. [25] and Ren et al. [26], enrichment of bioactive peptide from protein hydrolysates could be carried out using ultrafiltration method, which was employed and modified as ultrafiltration-centrifugation technique in present study. The digested peptide was fractionated into three groups according to molecular weight, i.e., 100-500, 500-1000, and 1000-3000 Da. The intact extract peptide was also treated with the same fractionation at the same time as a control test. The peptide yield and the DPPH• scavenging capacities of each group were detected. The results were illustrated in Table 2.

For peptide yields, it ranged from (4.9 ± 1.1)% to (6.4 ± 0.5)% and (5.7 ± 0.7) to (20.9 ± 2.1)% for undigested and digested sample with various molecular weight fractions, respectively (Table 2). An increase of four times of peptide with molecular mass of 500-1000 Da was detected during* in vitro* digestion, while other fractions slightly increased. Particularly, it was not statistically (*P* < 0.05) different in fractions with molecular mass of 100-500 Da for undigested and during digestion treatment. The significant increase of peptide with molecular mass of 500-1000 Da apparently agrees to the findings in literature, which reported that the fraction with molecular weight less than 1 kDa had the highest peptide yield for pepsin + pancreatin hydrolysates of pigeon pea protein [25].

For DPPH• assay, it ranged from (34.3 ± 2.9)% to (65.3 ± 8.5)% and from (35.2 ± 3.1)% to (81.2 ± 4.0)% for digested and undigested fractions, respectively. For digested and undigested samples, peptide with molecular weight of 1000-3000 Da demonstrated less DPPH• scavenging capacities, compared to fraction with molecular weight less than 1000 Da. This result was verified by Olagunju, Omoba, Enujiugha, Alashi, & Aluko [25], who observed that the highest scavenging activity against DPPH• was the digested fraction with molecular weight <1 kD of pigeon pea protein. In literature, it has been confirmed that peptides with low molecular weight exhibited higher antioxidative properties due to their smaller steric hindrance and the active center of short chain with hydrophobic and hydrophilic amino acid residues approaching easily to and reacting with free radicals, and ultrafiltration method also claimed to enrich these small molecules [5]. However, the DPPH• scavenging capacities of each fraction did not demonstrate the same variation trend with their yield, whether treated with digestion or not. The peptide digested with 500-1000 Da of molecular weight exhibited highest DPPH• scavenging capacities (*P *< 0.05), at (81.2 ± 4.0)%. No reports with similar fractionation were found in literature. It was inferred that the abundant antioxidant amino acid residues released from the huge protein might be respondent to the significant increase of peptide during digestion with molecular weight of 500-1000Da, while the fraction with 100-500 Da in undigested peptide displayed very high DPPH• scavenging capacities, (65.3 ± 8.5)%, the peptide yield of which was only (5.2 ± 0.3)%, only accounting for 25% of the former. High DPPH• scavenging capacities of peptide derived by intact extraction peptide were reported from* Chlorella* [12]. The puzzled problem was the digested fraction with 100-500 Da loss of the high DPPH• scavenging capacities during digestion. It was inferred that intact peptide with molecular weight of 100-500 Da was easily hydrolyzed into smaller pieces, losing bioactivity during digestion, and new members with less activity come from the larger peptide breaking.

### 3.5. Identification of Peptide Fractions with Excellent Antioxidant Capacity

**Figure 4:**
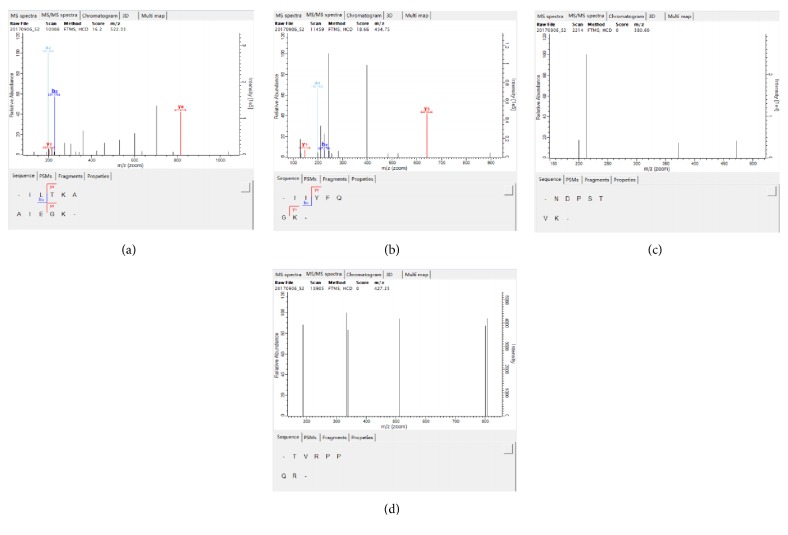
The second mass spectrogram of the identified peptides.

**Figure 5:**
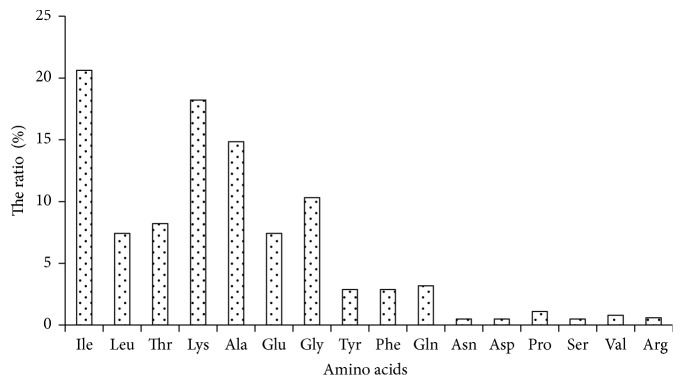
The ratio of amino acids in the novel peptides.

Liquid chromatography coupled to tandem mass spectrometry (LC-MS), as an effective tool, has been widely applied for analyzing various ingredients from biological samples, especially excellent for the sensitive analysis of the untargeted compounds. In this study, the digested 500-1000 Da fractions with highest DPPH• scavenging capacity were subjected to RPLC-Q Exactive HF mass spectrometer analysis for the identification of amino acid sequences and molecular mass responsible for antioxidant activity. The second mass spectrograms of the identified peptides were illustrated in Figure 4. Four major novel peptides were separated and identified, ranging from 7 to 10 amino acid residues in length and their amino acid sequences were found to be (a) Ile-Leu-Thr-Lys-Ala-Ala-Ile-Glu-Gly-Lys (1042 Da); (b) Ile-Ile-Tyr-Phe-Gln-Gly-Lys (867 Da); (c) Asn-Asp-Pro-Ser-Thr-Val-lys (759 Da); (d) Thr-Val-Arg-Pro-Pro-Gln-Arg (852 Da) (Figure 4). Delgado and the coworkers (2019) also obtained four small antioxidant peptides by gastrointestinal digestion coupled to LC-MS/MS from* Amaranth* proteins [45]. To analyze the relationship of structure and activity, the percentage of each amino acid in the total AAs from 4 peptides was investigated and illustrated in Figure 5. From Figure 5, a domination of several amino acids such as Ile, Lys, ala, Gly, Thr Leu, and Glu and a lack of some amino acids such as His, Met, Cys, and Trp were also found in many other antioxidant peptides [7]. And in the total amino acids, the high proportion of hydrophobic amino acids accounting for 56% was found which has been considered as the key factor in peptide ability to scavenge radicals. Moreover, some special amino acids such as Tyr, Thr, and Lys, having been reported to possess notable antioxidant properties, were also found in the novel peptides and account for 29.3% in total amino acids (Figure 5). In addition, basic amino acids were found such as Asn, Gln, and Arg in the identified peptides, which had been reported processing greater capacity to scavenge DPPH free radical than acidic or neutral peptides. Moreover, the identified dominant peptide also included the hydrophobic amino acid residues Ile and Leu at the N-terminus, which might be mainly responsible for the antioxidant capacity of the whole digested 500-1000 Da fraction.

## 4. Conclusions

In present study, the peptides from* Dunaliella salina *were extracted, digested by simulated* in vitro *gastrointestinal digestion, and fractionated to be optimized as the DPPH• scavenger. The results showed that DPPH• scavenging capacity of the peptide significantly increased due to simulated* in vitro *digestion. The peptide of 500-1000 Da fraction during digestion showed highest capacity, from which 4 peptides were identified. The results indicated that* Dunaliella salina* has potential to be a useful functional ingredient in food and nutraceutical industry for it can be processed in the human gastrointestinal environment to maximise the use of underutilised microalgae protein sources. In the next step, the key attention should be paid to further investigate other bioactivities of the peptide to deal with the chronic diseases.

## Figures and Tables

**Table 1:** The yield and DPPH• scavenging activity of non-digested or digested peptide.

Peptide fraction (Da)	Peptide yield (%)	DPPH• scavenging rate (%)
Non-digested	16.5 ± 1.5	46.8 ± 6.5
Digested	35.7 ± 2.9	86.5 ± 10.1

**Table 2:** The yield, DPPH• scavenging rate of different peptide fractions.

Peptide fraction (Da)	Peptide yields (%)		DPPH• scavenging rate (%)	
	Non-digestion	Digested	Non-digestion	Digested
100-500	5.2 ± 0.3	5.7 ± 0.7	65.3 ± 8.5	35.2 ± 3.1
500-1000	4.9 ± 1.1	20.9 ± 2.1	34.3 ± 2.9	81.2 ± 4.0
1000-3000	6.4 ± 0.5	9.1 ± 0.8	39.6 ± 6.3	45.5 ± 3.7

## Data Availability

The figures data used to support the findings of this study are included within the article.
